# A quality improvement initiative for achieving target tacrolimus concentration in renal transplant recipients: A quality improvement article,

**DOI:** 10.1097/MD.0000000000048443

**Published:** 2026-04-24

**Authors:** Ching-Yao Cheng, Cheng-Hsu Chen, Ming-Ju Wu, Hsiu-Mei Chen, Kuo-Hsiung Shu, Shang-Feng Tsai

**Affiliations:** a Department of Pharmacy, Taichung Veterans General Hospital, Taichung, Taiwan; b School of Pharmacy, China Medical University, Taichung, Taiwan; c Division of Nephrology, Department of Internal Medicine, Taichung Veterans General Hospital, Taichung, Taiwan; d Department of Post-Baccalaureate Medicine, College of Medicine, National Chung Hsing University, Taichung, Taiwan; e Department of Life Science, Tunghai University, Taichung, Taiwan; f PhD Program in Tissue Engineering and Regenerative Medicine, College of Medicine, National Chung Hsing University, Taichung, Taiwan; g Division of Nephrology, Department of Internal Medicine, Lin Shin Hospital, Taichung, Taiwan; h Division of Clinical Informatics, Department of Digital Medicine, Taichung Veterans General Hospital, Taichung, Taiwan.

**Keywords:** biopsy proved acute rejection (BPAR), intrapatient variability (IPV), mistake-proofing, multifaceted intervention, qualify improvement (QI), renal transplant, tacrolimus

## Abstract

Tacrolimus is a cornerstone immunosuppressive agent in solid organ transplantation due to its efficacy in preventing rejection. Achieving optimal tacrolimus levels remains challenging and limited studies have explored quality improvement (QI) initiatives aimed at optimizing tacrolimus trough levels. We conducted a QI initiative at Taichung Veterans General Hospital targeting tacrolimus level optimization in renal transplant recipients between January 2015 and January 2019. This multifaceted intervention included: physician reminders integrated into the Electronic Health Information System (EHIS) to monitor trough levels and variability, patient engagement through a mobile application displaying immunosuppressant levels, and automated tacrolimus concentration measurement to reduce ordering errors. Outcomes were descriptively evaluated before and after the interventions, including measurement accuracy, biopsy-proven acute rejection (BPAR) rates, and intrapatient variability (IPV), without formal statistical comparison. The implementation of automated tacrolimus measurement eliminated ordering errors, leading to an estimated cost saving of $78,911.3 over 2 years. A numerical decrease in BPAR incidence was observed, from 9% pre-intervention to 0% post-intervention. Tacrolimus intrapatient variability (IPV) also showed a decreasing trend, from 26.45% in 2019 to 18.7% in 2022. Physician reminders and patient engagement interventions contributed to improved monitoring but had a less pronounced impact on acute rejection rates. This study describes a structured QI initiative, including the automation of tacrolimus concentration measurements, and its association with improvements in therapeutic drug monitoring processes in renal transplant recipients. These findings suggest a potential role for mistake-proofing mechanisms in enhancing measurement accuracy and contributing to cost savings. Observed changes in rejection rates were descriptive and should be interpreted with caution given the small number of events.

## 1. Introduction

Tacrolimus is a calcineurin inhibitor and an essential immunosuppressive medication used primarily in solid organ transplant recipients.^[1]^ Due to its narrow therapeutic window, precise therapeutic drug monitoring (TDM) is critical to ensure effective treatment while minimizing potential toxicities. The drug’s metabolism is primarily mediated by the hepatic and intestinal cytochrome P450 (CYP) enzyme systems, particularly the CYP3A4 isoenzyme, with significant implications for individual variability in blood concentrations and dose requirements. In clinical practice, achieving target tacrolimus concentrations can be challenging due to the drug’s wide intra- and interindividual variability. Factors influencing tacrolimus pharmacokinetics include the patient’s genetic makeup,^[2-4]^ concomitant medications that affect enzyme activity,^[5]^ and physiological conditions, such as liver function.^[6]^ This variability underscores the importance of utilizing predictive models for tacrolimus concentration to optimize TDM and improve clinical outcomes, especially in the critical early days following transplantation when patients are at higher risk for adverse effects. Various strategies have been explored in recent years to improve the attainment of target tacrolimus levels. The use of prolonged-release tacrolimus formulations enables once-daily dosing, with the potential to enhance treatment adherence.^[7]^ Beyond trough levels, tacrolimus variability has also been reported to be associated with patient outcomes.^[8]^ The implementation of computerized dosing systems in renal transplant recipients has been shown to improve the achievement of target levels.^[9]^ Furthermore, recent studies have demonstrated promising results using machine learning algorithms to predict tacrolimus blood concentrations with greater accuracy compared to traditional pharmacokinetic models.^[10]^ However, several factors remain associated with tacrolimus trough levels.

The American College of Surgeons (ACS) Quality Improvement Case Study Repository serves as a valuable resource for hospitals engaged in quality improvement (QI) initiatives under the ACS Quality Programs.^[11-13]^ This repository houses a diverse array of QI projects developed by surgical clinical reviewers, cancer registrars, program directors, and other healthcare professionals. Each project is meticulously documented in accordance with the Quality Framework, offering a structured approach to understanding the planning, execution, data analysis, and key lessons derived from these initiatives.^[13,14]^ In the context of tacrolimus concentration management, recent studies underscore the importance of process optimization within laboratory settings.^[15,16]^ One study demonstrated that adjusting immunosuppressant collection and analysis workflows significantly improved the laboratory’s ability to meet physician-requested reporting times for tacrolimus levels.^[15]^ By applying Lean methodologies, laboratories can effectively identify workflow bottlenecks and implement standardized processes to enhance specimen processing efficiency.^[16]^ Therefore, maximizing the number of samples loaded into a single rack during tacrolimus testing streamlines the workflow and improves turnaround times for results.^[16]^ These process optimization initiatives not only exemplify a strong commitment to continuous quality improvement but also contribute to enhanced patient outcomes through more timely and accurate medication management. There is a paucity of studies examining other aspects of the workflow aimed at improving the attainment of target tacrolimus trough levels through QI initiatives.

Several factors remain associated with achieving target tacrolimus trough levels, including patient engagement, the accuracy of physician-ordered trough level measurements, and reminder systems integrated into outpatient clinic workflows. To date, there have been limited studies investigating the impact of these factors on tacrolimus level management. In this study, we aimed to share our experience in improving the attainment of target tacrolimus levels by addressing key aspects of patient care, including patient engagement, the accuracy of physician-ordered measurements, and outpatient clinic system reminders.

## 2. Materials and methods

We present the process and outcomes of our QI collaborative initiative aimed at optimizing the target levels of tacrolimus in renal transplant recipients. This study also evaluates projected outcomes associated with these interventions from January 01, 2015 to January 01, 2019. A multifaceted intervention was introduced, and data were collected before and after its implementation. Our primary objective was to maintain tacrolimus levels within the recommended therapeutic range. The intervention was developed through a systematic review of all relevant stakeholders, including both healthcare providers and patients. A structured, multidisciplinary approach was employed to optimize tacrolimus target levels. All clinical outcomes were thoroughly assessed pre- and post-intervention to evaluate the impact of the QI initiative. Our findings provide insights into the effectiveness of a structured QI approach in improving tacrolimus level management in renal transplant recipients.

### 2.1. Definition of population and study design

This QI study focused on renal transplant recipients at our institution. We retrospectively reviewed patients who underwent renal transplantation at our center between 2013 and 2022. A multifaceted intervention was implemented, including: the incorporation of tacrolimus trough levels and variability monitoring for physicians since January 01, 2017, a patient self-monitoring system for trough levels via a mobile application to enhance patient empowerment since January 01, 2018, and automated tacrolimus concentration measurement introduced since January 01, 2019. To assess the effectiveness of these interventions, clinical outcomes were evaluated before and after their implementation. This study was approved by the Institutional Review Board of Taichung Veterans General Hospital (approval number: CW17045A). The requirement for patient consent was waived as the study utilized de-identified data and posed minimal risk to participants.

### 2.2. Outcomes and data collection

To evaluate the impact of this QI initiative on renal transplant recipients, we systematically collected outcome data from January 01, 2013 to December 31, 2022. The primary outcome was the accuracy of immunosuppressant trough level measurements. The secondary outcome was the incidence of acute rejection in new renal transplant recipients within 2 weeks and 1-year posttransplant. The other secondary outcome was intra-patient variability (IPV) of tacrolimus trough level, expressed as a percentage, which was defined by the variability in tacrolimus trough levels during the 6 to 12 months following transplantation. In clinical practice, tacrolimus levels tend to become more stable approximately 6 months after renal transplantation, usually within the range of 6 to 8 ng/mL. The intra-patient variability (IPV) index (FK%) was calculated as the coefficient of variation (CV), using the formula: CV (%) = (standard deviation/ mean) × 100. By providing real-time visualization of serial trough levels and tacrolimus variability, this EHIS integration enhances clinical decision-making and supports more precise immunosuppressant dosing for renal transplant recipients. Only patients with at least 3 measurements of FK levels were included for the analysis of IPV, and all FK levels obtained between 6 and 12 months after renal transplantation were analyzed.

For accuracy of immunosuppressant trough level measurements, we collected zero-concentration trough levels of sirolimus, everolimus, tacrolimus, and cyclosporine from 2017 to 2018. Errors in trough level measurements were classified as “definite” incorrect orders, defined as cases where no immunosuppressant prescription was documented within 4 months prior to the trough level measurement, and “possible” incorrect orders, defined as cases where an immunosuppressant prescription was recorded within 4 months prior to the measurement. Additionally, the financial cost associated with unnecessary concentration measurements due to definite incorrect orders was estimated. For the secondary outcome, the yearly incidence of biopsy-proven acute rejection within 2 weeks and 1-year posttransplant was analyzed and compared from 2013 to 2022.

### 2.3. Statistical analysis

This study was conducted as a QI initiative; therefore, analyses were primarily descriptive in nature. Categorical variables, including the proportion of incorrect or potentially incorrect orders, are presented as counts and percentages. Continuous variables, such as IPV of tacrolimus trough levels, are summarized using mean values across years. Cost data were aggregated and reported as total expenditures associated with incorrect and potentially incorrect orders. For clinical outcomes, including BPAR, yearly incidence rates were calculated based on the number of events and newly enrolled transplant recipients per year. Given the small number of BPAR events (generally < 10 cases/yr), formal statistical comparisons were not emphasized, as such analyses may yield unstable estimates and potentially misleading inferences. Therefore, these data are presented descriptively, and observed changes over time should be interpreted with caution. Temporal trends in IPV were assessed descriptively across study years without formal hypothesis testing.

## 3. Results

### 3.1. Enlisting a stakeholder mapping

**Figure 1.**
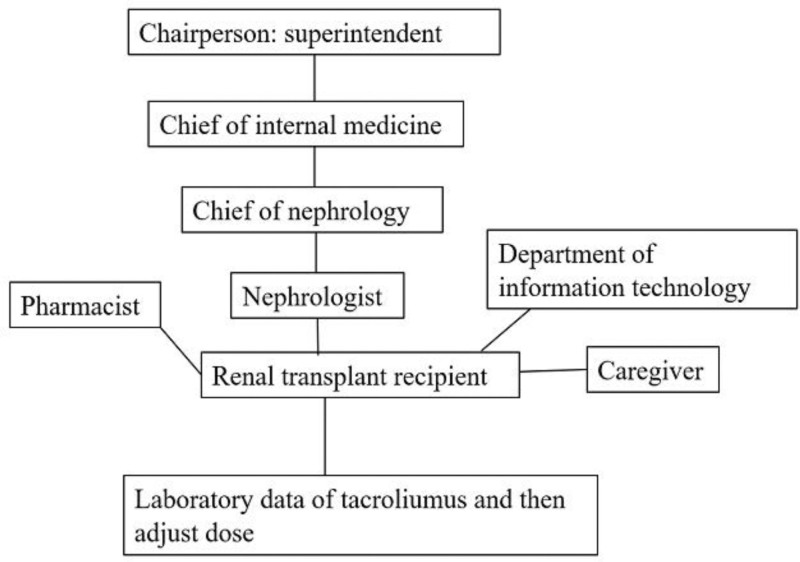
Stakeholder map of quality improvement for tacrolimus concentration.

To systematically implement the QI initiative, we first conducted a stakeholder mapping to identify individuals and groups who could influence the project (Fig. 1). Stakeholders were then categorized into distinct groups, including nephrologists, pharmacists, patients, patients’ caregivers, and information technology (IT) staff, with their interrelationships outlined. The central focus of the mapping was the quality element, ensuring that all stakeholders contributed to the optimization of trough level of immunosuppressants.

Nephrologists were responsible for accurately ordering immunosuppressant trough level measurements for renal transplant recipients. The trough levels were subsequently available for review by doctors in the hospital’s Electronic Health Information System (EHIS). Patients and their caregivers were able to access this data remotely and discuss the results with their physicians and pharmacists. All system design and modifications were conducted by the hospital’s IT staff. This QI initiative was supported by the superintendent of our hospital.

### 3.2. Intervention 1 – physician reminder: trough levels (FK) and variability of tacrolimus (FK%) in the EHIS system for doctors

**Figure 2.**
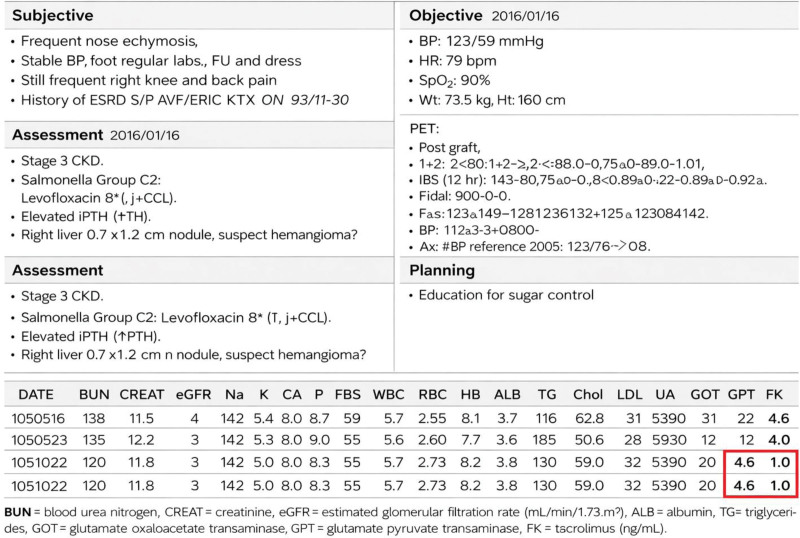
Trough levels (FK) and variability of tacrolimus (FK%) in the EHIS system for doctors since 2017. EHIS = Electronic Health Information System, FK = tacrolimus.

Since January 01, 2015, the IT department has implemented a serial trough level monitoring system within the EHIS (Fig. 2). This system enables physicians to access the 3 most recent consecutive trough levels of tacrolimus (FK), sirolimus, and everolimus directly from the patient’s EHIS homepage.

### 3.3. Intervention 2 – patient engagement: display of trough levels of immunosuppressants for patients in the APP

**Figure 3.**
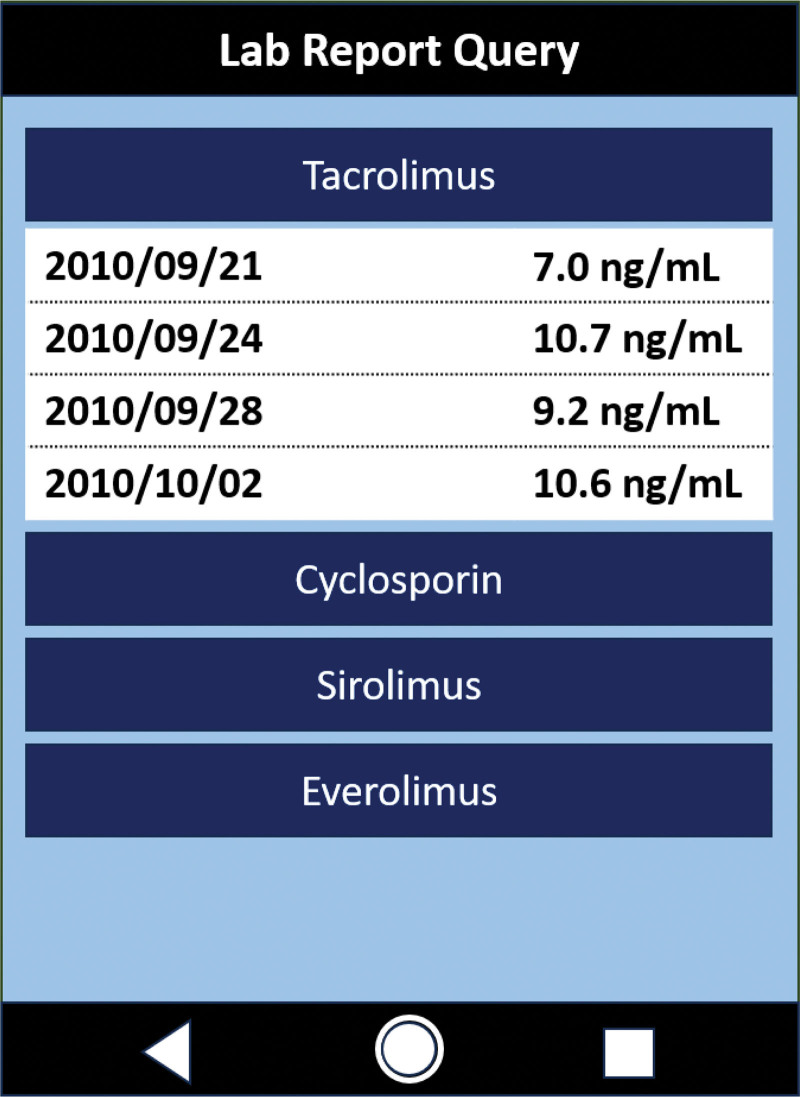
Display of trough levels of immunosuppressants for patients in the APP since 2018. APP = mobile application.

Since January 01, 2018, a query system has been integrated into our institution’s mobile application to enhance patient and caregiver engagement (Fig. 3). Developed by the IT department, this system allows patients to access their immunosuppressant trough levels after logging in. The data are presented as a cumulative trough level report, providing a longitudinal overview of immunosuppressant levels. By enabling patients to monitor their trough levels independently, this system facilitates shared decision-making and encourages more informed discussions with physicians and pharmacists regarding immunosuppressant management.

### 3.4. Intervention 3 – automatic measurement: automatic order for measuring trough level of immunosuppressants

**Figure 4.**
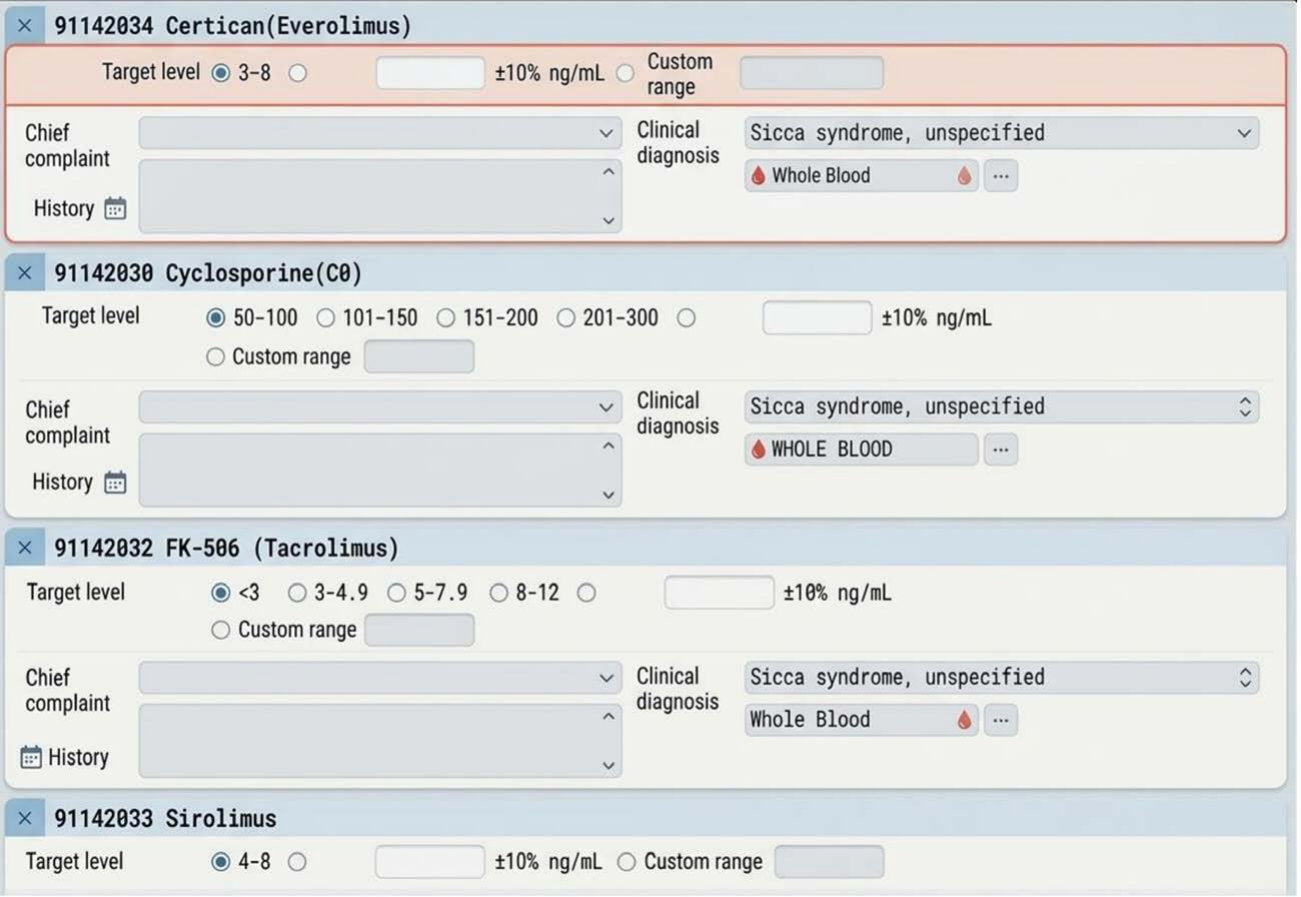
Automatic order for measuring trough level of immunosuppressants since 2019.

Since January 01, 2019, to prevent erroneous orders for immunosuppressant trough level measurements, the IT department implemented an automated measurement system (Fig. 4). Within the examination ordering function, the system automatically links trough level measurements to the prescribed immunosuppressants, ensuring that physicians do not order trough level tests for patients who are not receiving the corresponding medication. Additionally, this system allows physicians to manually adjust the target trough level as needed, providing greater flexibility in individualized immunosuppressant management.

### 3.5. Incorrect orders for immunosuppressants

Within the 2 years preceding the intervention 3, the number of definite and possible incorrect orders for all 4 immunosuppressants is summarized in Table 1. During this period, a total of 137 definite incorrect orders and 4130 possible incorrect orders were identified across all immunosuppressants. Among them, tacrolimus measurement was the most frequently associated with ordering errors, accounting for 97 definite incorrect orders and 709 possible incorrect orders. Everolimus measurement was the second most affected, with 22 definite incorrect orders and 563 possible incorrect orders.

**Table 1:** Data on 0 concentration trough levels of sirolimus, everolimus, tacrolimus, and cyclosporine from 2017 to 2018.

	Prescription of immunosuppressants for this trough level measurement within 4 mo		Total
	No (definite incorrect orders)	Yes (possible incorrect orders)	
Case numbers	93 (23.19%)	308 (76.01%)	401
Measurement times	137 (8.74%)	1430 (91.26%)	1567
According to medication			
Cyclosporin	8 (7.5%)	98 (92.5%)	106
Tacrolimus	97 (12.0%)	709 (88.0%)	806
Sirolimus	10 (14.3%)	60 (85.7%)	70
Everolimus	22 (3.8%)	563 (96.2%)	585

The financial impact of incorrect immunosuppressant measurement orders is summarized in Table S1, Supplemental Digital Content, https://links.lww.com/MD/R733. The wasted cost due to definite incorrect orders in 2017 and 2018 amounted to $6513.4 USD, while possible incorrect orders resulted in a wasted expenditure of $72,397.9 USD. In total, the estimated financial loss due to incorrect orders reached $78,911.3 USD over the 2-year period.

Following the implementation of the automated ordering system for immunosuppressant trough level measurements, no more incorrect orders have been recorded.

### 3.6. Yearly incidence of biopsy-proven acute rejection within 2 weeks and 1 year posttransplant from 2013 to 2022

The yearly incidence of BPAR is summarized in Table S2, Supplemental Digital Content, https://links.lww.com/MD/R733. The annual number of newly enrolled renal transplant recipients ranged from 22 to 66, with 0 to 6 BPAR events per year, corresponding to incidence rates between 0% and 13%. Across the study period, the incidence of BPAR showed year-to-year variability without a consistent pattern. Following intervention 1, the incidence ranged from 10% to 13% between 2015 and 2017. After the implementation of intervention 2 in 2018, the incidence was 9%. In 2019, after the introduction of intervention 3, no BPAR events were observed. In subsequent years, BPAR incidence varied between 5% and 10%. Given the small number of events, these findings are descriptive and should be interpreted with caution.

### 3.7. Annual incidence of intrapatient variability in tacrolimus trough levels (IPV)

The annual IPV of tacrolimus trough levels is presented in Table S2, Supplemental Digital Content, https://links.lww.com/MD/R733. Prior to any interventions, the IPV was approximately 21.1% in 2014. Following the implementation of intervention 1, IPV increased to 24.8% in 2015 and further to 33.5% in 2016. However, a declining trend was observed after the introduction of intervention 2 in 2018. Subsequently, with the initiation of intervention 3 in 2019, IPV decreased to 26.45% in 2019 and 24.4% in 2020. This downward trend continued, with IPV falling below 20%, reaching 18.7% by 2022.

## 4. Discussion

Targeted QI interventions focusing on tacrolimus level monitoring remain underexplored. This study represents the first QI initiative aimed at enhancing the accuracy of tacrolimus trough level monitoring in renal transplant recipients. Through the implementation of 3 multifaceted interventions, we successfully eliminated errors in tacrolimus concentration measurements, achieving a zero-error rate. Additionally, the initiative resulted in an estimated cost savings of approximately $78,911.3 over 2 years. Changes in the incidence of BPAR and mean IPV were observed among new renal transplant recipients over the study period. We recommend that institutions managing renal transplant patients adopt our QI framework to improve care quality.

In this study, the incidence of acute rejection remained similar following intervention 1 (physician reminders), with rates changing from 10% to 13%. A slight reduction was observed after intervention 2 (patient engagement), decreasing from 10% to 9%. However, a dramatic reduction in acute rejection incidence was achieved after intervention 3, with rates declining from 9% to 0%. In our institution, the implementation of physician reminders was not significantly associated with a reduction in the incidence of acute rejection. However, this does not diminish the importance of tacrolimus concentration monitoring. We believe this finding is due to the fact that, even before the introduction of the EHIS reminder system (serial tacrolimus trough level and variability), nephrologists and pharmacists in our institution were already highly attentive to renal transplant recipients. We routinely monitored patients’ tacrolimus trough levels and calculated variability manually. While the reminder system did not significantly alter rejection rates, it provided greater convenience, allowing us to manage patients more efficiently. This reasoning also explains why intervention 2 (patient engagement) was not significantly associated with a reduction in acute rejection incidence. In our institution, nephrologists and pharmacists closely monitor tacrolimus levels and make dose adjustments. However, patient engagement remains crucial for other outcomes, such as infection prevention and medication adherence. Among the 3 interventions, the most impactful in our setting was intervention 3: automated prescription for tacrolimus concentration monitoring. This intervention not only reduced costs but also ensured that patients had tacrolimus trough level assessments at every outpatient visit. With a 100% correct measurement rate, nephrologists and pharmacists could adjust the drug dosage promptly and accurately. Thus, in our institution, automated tacrolimus monitoring played a pivotal role in optimizing immunosuppressive therapy and improving patient management.

Mistake-proofing mechanisms were implemented to reduce measurement errors in tacrolimus trough level assessments, representing the most critical intervention in this QI initiative. According to the landmark review To Err is Human, it is estimated that up to 98,000 deaths occur annually in hospitals due to medical errors.^[17]^ These errors contribute to substantial avoidable healthcare costs, exceeding $17 billion annually in direct costs in the United States alone.^[18]^ Patient identification errors have been reported in approximately 0.005% to 1% of laboratory samples.^[19]^ Furthermore, up to 62% of diagnostic errors occur during the pre-analytical phase, with an additional 23% occurring in the post-analytical phase.^[20,21]^ Despite these findings, no studies to date have specifically addressed the incidence of order errors related to immunosuppressant concentration measurements. Our study demonstrates that order errors – the initial step in the laboratory process – play a pivotal role in the success of our QI initiative.

To date, no studies have reported on the automation of tacrolimus concentration measurements. The likelihood of kidney transplant patients undergoing a repeat tacrolimus level assessment after a lab order error or incorrect blood draw depends on several factors, including institutional protocols, the patient’s clinical condition, and the timing of error identification. In most cases, if the error is detected before the patient takes their next dose, an additional blood draw may be arranged. However, if the error is identified after the patient has taken their medication, accurate trough level measurement becomes impossible, and a repeat blood draw is typically not performed. Based on clinical experience, most patients do not undergo repeat blood draws unless the error is identified on the same day and the data is critical for therapeutic decision-making. Furthermore, since patients are required to take their tacrolimus dose following an incorrect blood draw, obtaining an accurate trough level for that day becomes unfeasible. These challenges highlight the importance of laboratory automation. Published studies have demonstrated that total laboratory automation enhances laboratory productivity and efficiency.^[22]^ Additionally, automation has been shown to reduce random errors, improve time management, and optimize bioanalytical parameters.^[23]^ By minimizing pre-analytical and analytical errors, laboratory automation contributes to improved patient safety and supports institutions in achieving their quality and safety goals. Our study is the first to demonstrate that automating the ordering process for tacrolimus concentration measurements significantly improves patient outcomes, underscoring the critical role of automation in optimizing care for kidney transplant recipients.

This study has several limitations. Primarily, it is a single-center study with a limited sample size, which may affect the generalizability of the findings. However, the primary objective of this study was to share our experience with a QI initiative focused on optimizing tacrolimus trough level monitoring. Due to the limited case number (<10 cases/yr for patients with BPAR), we are unable to perform meaningful statistical analyses. The statistical power is extremely limited and any formal test may yield misleading results. Therefore, we have presented descriptive statistics instead, allowing only for observation of trends. Secondly, with respect to the outcome of BPAR, several important factors – such as donor type (living vs deceased), immunization status, age, sex, and HLA donor–recipient mismatches – were not accounted for in the analysis and may have influenced the results. The interventions implemented in this study – including physician reminders, patient engagement, and automated measurement – were associated with improvements in process-related outcomes, particularly in reducing incorrect orders. As this was a quality improvement initiative, the study was not designed to evaluate clinical outcomes such as BPAR. Despite these limitations, the findings suggest that this QI framework may be adaptable to other healthcare settings to support the management of renal transplant recipients. Further studies with larger sample sizes and multicenter designs are needed to validate these observations.

## 5. Conclusion

This study describes a QI initiative aimed at optimizing tacrolimus trough level management in renal transplant recipients. Through a multifaceted intervention approach, improvements in process-related outcomes and cost savings were observed. Changes in BPAR incidence and tacrolimus IPV were also noted; however, these findings are descriptive and should be interpreted with caution given the small number of events. Among the interventions, the implementation of automated tacrolimus measurement may represent an important mistake-proofing mechanism within the medication monitoring process.

## Acknowledgments

With thanks to division of clinical informatics, department of digital medicine, Taichung Veterans General Hospital.

## Author contributions

**Conceptualization:** Ching-Yao Cheng, Ming-Ju Wu, Shang-Feng Tsai.

**Data curation:** Ching-Yao Cheng, Cheng-Hsu Chen, Ming-Ju Wu, Hsiu-Mei Chen, Kuo-Hsiung Shu, Shang-Feng Tsai.

**Formal analysis:** Shang-Feng Tsai.

**Funding acquisition:** Shang-Feng Tsai.

**Investigation:** Shang-Feng Tsai.

## Supplementary Material
