# Supplementary material for: A quality improvement initiative for achieving target tacrolimus concentration in renal transplant recipients: A quality improvement article,
Source: Medicine (Baltimore). 2026 Apr 24;105(17):e48443. doi: 10.1097/MD.0000000000048443 (PMC13124434; doi:10.1097/MD.0000000000048443)
Supplement: Supplementary file 1 [file medi-105-e48443-s001.pdf]

Supplementary data

Table 1. The financial cost associated with incorrect orders for concentration trough level measurements of sirolimus, everolimus, tacrolimus, and cyclosporine from 2017 to 2018.

	The total cost per measurement (US dollars)	Prescription of immunosuppressants for this trough level measurement within 4 months		total
		No (definite incorrect orders)	Yes (possible incorrect orders)	
Cyclosporin	13.3	106.4	1303.4	1409.8
Tacrolimus	45	4365	31905	36270
Sirolimus	66.7	667	4002	4669
Everolimus	62.5	1375	35187.5	36562.5
Subtotal	187.5	6513.4	72397.9	78911.3

Supplementary data

Table 2. Yearly incidence of biopsy-proven acute rejection within 2 weeks and one year post-transplant, and intra-patient variability of tacroliumus trough levels during the 6–12 months post-transplantation from 2013 to 2022

Year	Case numbers	All newly enrolled patients during the current year	Incidence (%)	Mean IPV (%)
2013	2	40	5	24.7%
2014	1	66	2	21.1%
2015 (start intervention 1)	6	59	10	24.8%
2016	8	62	13	33.5%
2017	4	40	10	29.0%
2018 (start intervention 2)	5	55	9	28.8%
2019 (star intervention 3)	0	40	0	26.4%
2020	3	34	9	24.4%
2021	4	40	10	20.2%
2022	1	22	5	18.7%
